# The Chimeric LFC and DCIA Flap in Combined Mandibular and Condylar Head and Neck Reconstruction—A Case Series

**DOI:** 10.3390/jcm13123613

**Published:** 2024-06-20

**Authors:** Christoph Steiner, Maximilian Neubert, Gian B. Bottini, Shinnosuke Nogami, Katharina Zeman-Kuhnert, Alexander Gaggl

**Affiliations:** 1 Department of Oral and Craniomaxillofacial Surgery, Paracelsus Medical University Salzburg, Müllner Hauptstraße 48, 5020 Salzburg, Austria; ch.steiner@outlook.com (C.S.); neubert.maximilian@gmail.com (M.N.); g.bottini@salk.at (G.B.B.); s-nogami@dent.tohoku.ac.jp (S.N.); k.zeman-kuhnert@salk.at (K.Z.-K.); 2 Division of Oral and Maxillofacial Surgery, Department of Oral Medicine and Surgery, Tohoku University Graduate School of Dentistry, Sendai 980-8575, Miyagi, Japan

**Keywords:** lateral femoral condyle flap, DCIA flap, chimera flap, mandibular reconstruction, TMJ reconstruction, plastic and reconstructive surgery, maxillofacial surgery

## Abstract

**Background**: Defects of the ascending ramus of the mandible, including the condylar head and neck or the whole temporomandibular joint (TMJ), are difficult to reconstruct. Reconstruction is mainly based on the use of alloplastic joint prosthesis, costochondral grafting, distraction osteogenesis of the dorsal part of the mandibular ramus, or osseous microvascular flaps of various origin. With the objective of developing a method that overcomes the restrictions of these methods, we recently introduced a sequential chimeric flap consisting of a lateral femoral condyle flap (LFC) and deep circumflex iliac artery flap (DCIA) for reconstruction of up to half of the mandible and the condylar head and neck. **Methods**: The chimeric flap was used in four patients with the following diagnoses: therapy-refractory osteomyelitis, extended recurrent odontogenic keratozyst, Goldenhar syndrome, and adenocarcinoma of the parotid gland. After a diagnostic workup, LFC and DCIA flaps were harvested in all patients and used in a sequential chimeric design for the reconstruction of the mandibular body and condylar head and neck. **Results**: Follow-up from at least 24 months up to 70 month after surgery showed a successful reconstruction in all four patients. The LFC provided a cartilaginous joint surface, allowing for a satisfactory masticatory function with a stable occlusion and unrestricted mouth opening and preserved or regained lateral and medial excursions in all patients. The DCIA allowed for a bony reconstruction anatomically resembling a non-atrophied mandibular body. No flap-related complications were observed. **Conclusions**: The sequential chimeric LFC and DCIA flap is an appropriate method for reconstructing up to half of the mandible and the condylar head and neck. It is suitable in cases where alloplastic joint replacement cannot be used or where other methods have failed. Due to the necessity of harvesting two flaps, the burden of care is increased, and a careful indication is required. The technique is reserved for maxillofacial surgeons who have already gained significant experience in the field of microsurgery.

## 1. Introduction

In tumor and osteonecrosis patients and patients with congenital malformations, maxillofacial surgeons are confronted with complex combined defects. Particularly, defects of the ascending ramus of the mandible including the condylar head and neck or the whole temporomandibular joint (TMJ) are difficult to reconstruct. Replacement of the condylar head and neck in TMJ reconstruction is mainly based on the use of alloplastic joint prosthesis (with or without the fossa component), costochondral grafting, distraction osteogenesis of the dorsal part of the mandibular ramus, or osseous microvascular flaps of various origin. Joint prostheses, nowadays usually as patient-specific implants, are, by comparison, a relatively simple method for reconstruction with good predictable results [1]. However, their application is restricted in cases with large defects; in infected sites, such as in cases of osteomyelitis; in young patients; post-irradiation; and in cases of allergies to the prosthesis materials. In these cases, an alternative treatment has to be performed [2,3,4].

With the objective of developing a method that overcomes these restrictions, we recently introduced a sequential chimeric flap consisting of a lateral femoral condyle flap (LFC) and deep circumflex iliac artery flap (DCIA) for the reconstruction of up to half of the mandible and the condylar head and neck. In this paper, we present this new flap combination in a case series of four patients.

## 2. Materials and Methods

We used the described chimeric flap in four patients (two females and two males) between 2016 and 2021 for simultaneous mandibular and condylar head reconstruction. The patients were 59, 63, 17, and 51 years old (Table 1). The primary diagnoses were therapy-refractory osteomyelitis (*n* = 1), extended recurrent odontogenic keratocyst involving the whole mandibular ramus and the condylar head (*n* = 1), Goldenhar syndrome (*n* = 1), and parotid gland adenocarcinoma (*n* = 1). The follow-up period was between 24 and 70 months (mean, 49 months). Written informed consent was obtained from all patients before surgery. 

### 2.1. Diagnostic Workup and Planning

**Figure 1:**
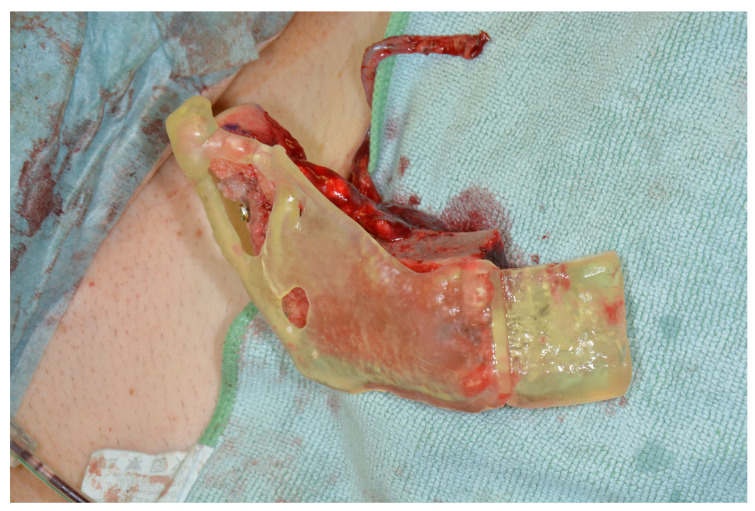
In-house printed 3D cutting guide for mandibular resection and template for the assembly of the sequential chimeric LFC and DCIA flap.

Computed tomography (CT) scans with a slice thickness of 0.5 mm or less of the entire mandible and midface were performed for the initial evaluation and for planning. The DICOM datasets were further processed using the PROPLAN CMF software (Materialise, Leuven, Belgium). Three-dimensional models and cutting guides were created (Figure 1). If necessary, the opposite side was mirrored for this purpose. Models and cutting guides were printed in-house using Form 2 and Form 4b 3D printers (Formlabs, Berlin, Germany). Finally, the models were sterilized and brought to the operating room. 

### 2.2. Surgical Procedure

Surgery was performed using a two-team approach. The first team prepared the recipient bed by performing a combined Risdon and preauricular approach to expose both the mandible and the TMJ. After finishing the primary surgery (i.e., mandibular resection, tumor resection,…), the recipient vessels were prepared. The articular disc was left in place, if present. A passive mouth opening of at least 40 mm was affirmed. Then, maxillomandibular fixation was performed in centric occlusion. In this position, the 3D models and cutting guides were checked for correctness.

Simultaneously, the second team first chose the side for harvesting the DCIA flap based on the extent and form of the mandibular reconstruction. Then, according to Grinsell and Cato-Smith, the anterior superior iliac spine and the course of the inguinal ligament were marked, and the femoral artery was palpated [5]. The surgical site was opened approximately 1 cm above the inguinal ligament. Dissection was carried out layer by layer down to the fascia of the external oblique muscle, which was then incised and dissected up to the fascial sheath of the transversus abdominis muscle. Here, the deep circumflex iliac artery (DCIA) and vein were identified. Initially, the ascending branch was dissected for a length of approximately 5 cm for supplying the femoral condyle later on. It was clipped and severed. Subsequently, the longitudinal branch was followed for a length of approximately 6.5 cm and clipped distally. Subperiosteal lateral dissection of the iliac crest was then performed, followed by osteotomy using a Piezosurgery device, resulting in a DCIA flap measuring on average 4 cm at the spine and 2.5 cm laterally, with a length according to the mandibular defect. The DCIA flap was for the time being left in situ.

**Figure 2:**
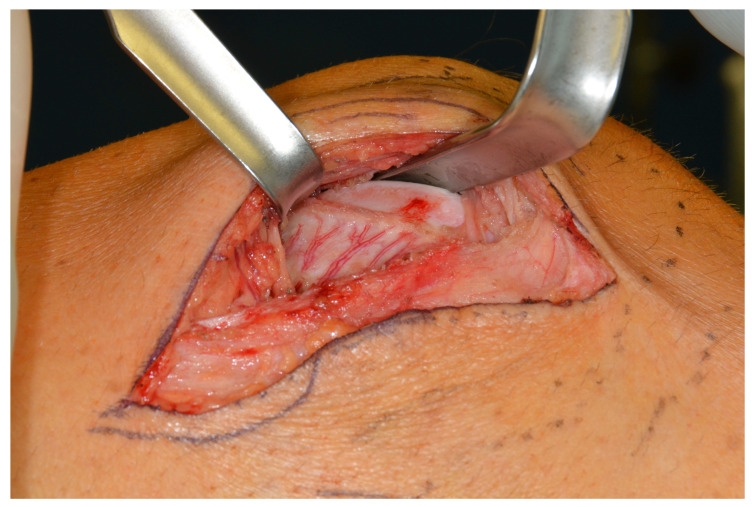
Harvesting the LFC.

We switched to the lateral femoral condyle (for details also see Enzinger et. al., 2018) [6]. First, the patella, knee joint space, and insertion of the iliotibial band were marked. The surgical site was opened above the perforator, penetrating the iliotibial band, and the perforator was dissected. Subsequently, the iliotibial tract was split longitudinally, and the perforator was followed into depth to its origin from the superior lateral genicular artery. The periosteal branches were identified and followed to the anterior pole of the knee joint, the joint capsule was incised, and the cartilage of the lateral femoral condyle was exposed. Dissection was continued as well into the area of the popliteal artery and vein (these of course had to be left intact) to gain enough pedicle length (Figure 2). 

**Figure 3:**
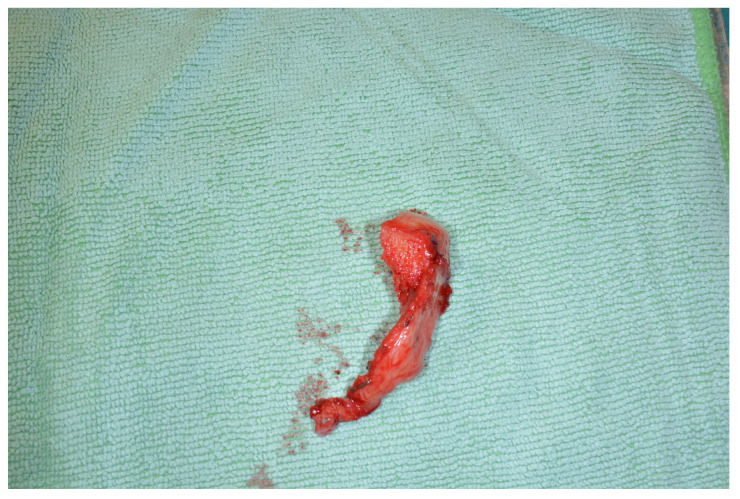
The osteochondral LFC.

After isolating the vascular pedicle, the cartilage was incised according to the cutting guide, and the bony cuts were performed using a Piezosurgery device and chisels. The cartilage portion was osteotomized, allowing for reconstruction of a neo-condyle measuring about 1 by 1.5 cm (Figure 3).

**Figure 4:**
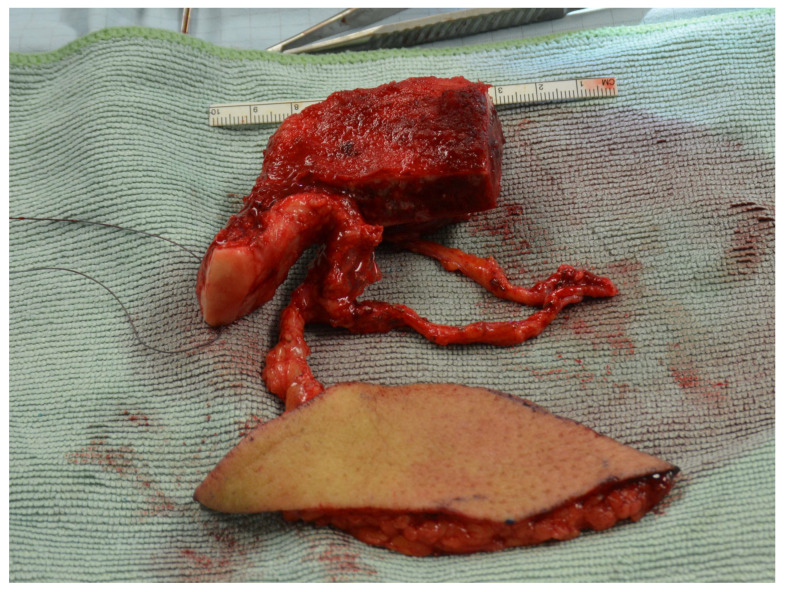
A skin perforator flap harvested together with the LFC for concomitant skin replacement after extended resections.

A skin-perforator flap can be included in the preparation, e.g., in cases of tumor resection, including the skin (Figure 4). 

Now, the supplying vessels were clipped and cut, and the transplant was transferred to the DCIA area. Wound closure in the knee area included thoroughly suturing the joint capsule and the iliotibial band with resorbable sutures and placement of two Redon drains, one placed within the joint space.

**Figure 5:**
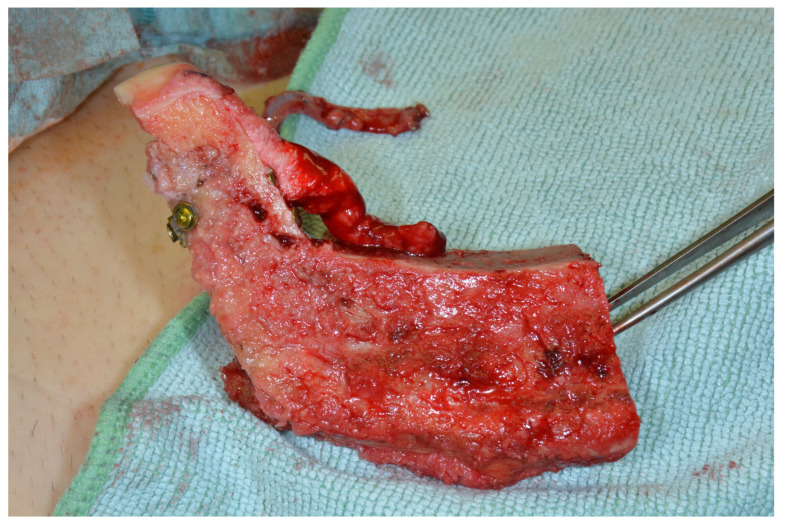
The sequential chimeric LFC and DCIA flap ready for transfer to the recipient site. Note the DCIA replacing the body and parts of the ramus of the mandible and the LFC replacing the condylar neck and head.

**Figure 6:**
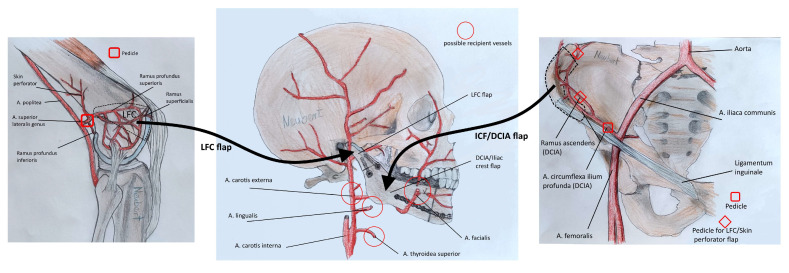
The sequential chimeric LFC and DCIA flap: an overview of the bony and vascular anatomy.

Now, we switched to the DCIA area. Here, the femoral condyle was adapted to the DCIA flap, becoming the future mandibular head and neck (Figure 5). The two parts were connected using three lag screws or miniplates for initial stability and simplifying the adaption of a reconstruction plate later on. Subsequently, microanastomoses were performed between the ascending branch and the superior lateral genicular artery as well as between the larger of the two accompanying veins of both flaps. Now, pulsations were observed up to the periphery of the femoral transplant, with bleeding from the cancellous bone area. After double vascular pedicle clipping, the chimeric flap was detached and transferred to the face. Wound closure was performed. Figure 6 gives an overview of the procedure.

**Figure 7:**
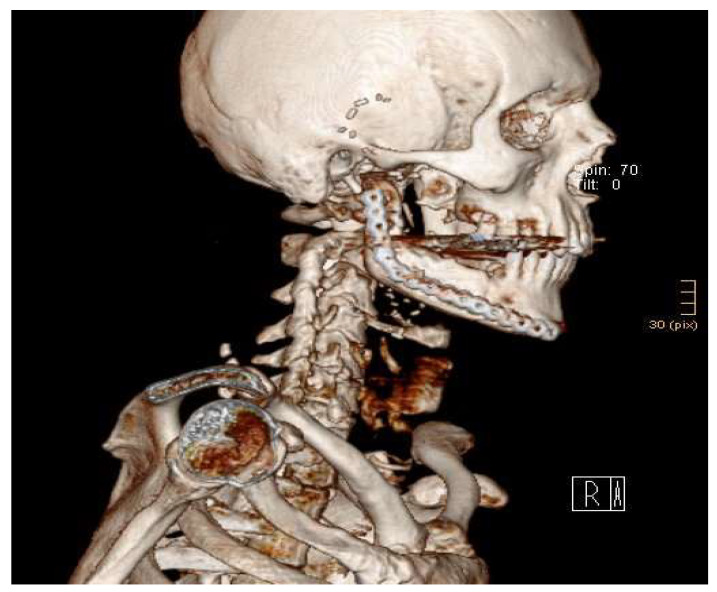
The 3D reconstruction of a CT scan showing an anatomically correct reconstruction of the right mandible up to the condylar head with the sequential chimeric LFC and DCIA flap.

Now, the chimeric flap was fixed to the remaining mandible using a reconstruction plate for sufficient stability (Figure 7). 

The joint capsule—when present—was fixed to the neo-condyle using four PDS sutures. The articular disc can be attached to a microplate on the neo-condyle. Microanastomoses were performed between the facial artery (if present; especially in tumor surgery, other vessels might have to be used) and the deep circumflex iliac artery, as well as the facial vein and the deep circumflex iliac vein, as end-to-end anastomoses. Before wound closure, the perfusion of the chimeric flap was controlled clinically and by a Doppler probe. Low-molecular-weight heparin was administered after completion of the anastomoses and continued every 24 h for 14 days. Maxillomandibular fixation was released, and the occlusion as well as mouth opening, laterotrusion, and mediotrusion were checked.

**Figure 8:**
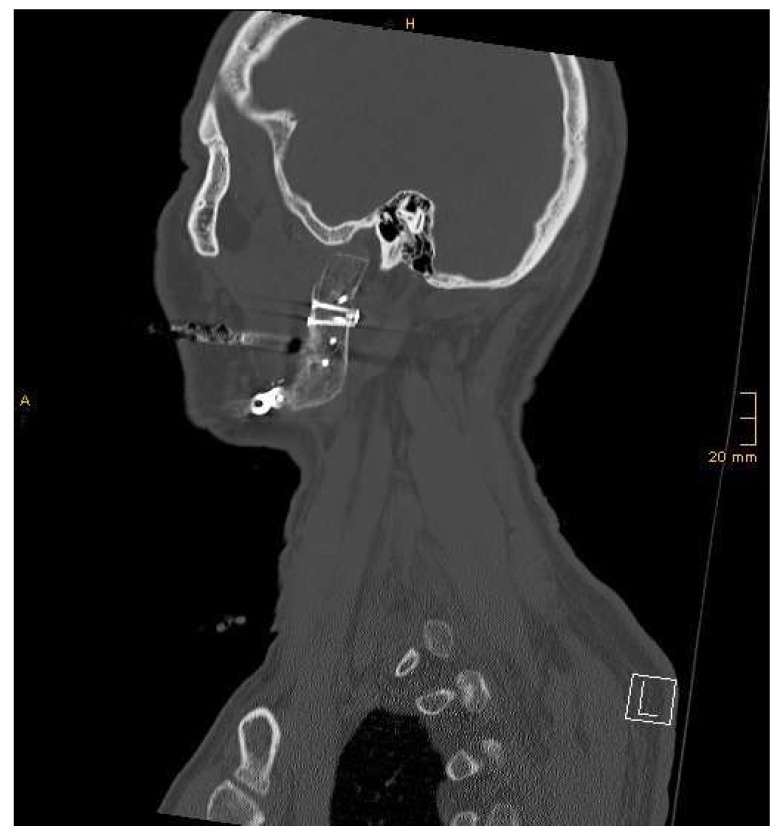
Sagittal CT view of the LFC in relation to the articular fossa, showing a good position.

All patients were admitted to the intensive care unit postoperatively for at least the first 24 h. The systolic blood pressure was maintained over 100 mmHg. Flap perfusion was affirmed every 2 h for 48 h with a handheld Doppler probe. Intra- or postoperative CT scans were performed directly in the operating theatre using the Loop-X Mobile Imaging Robot (Brainlab, Munich, Germany) (Figure 7 and Figure 8).

### 2.3. Postoperative Management

All patients received intravenous antibiosis with ampicillin/sulbactam 3 g (clindamycin 600 mg in case of penicillin allergy) every 8 h for up to 5 days. A liquid then soft diet is prescribed for 4–6 weeks post-surgery. Physiotherapy is initiated in the first week after surgery for general mobilization with following instructions for the physiotherapists: full weight-bearing, adapted to pain. Hip: use of an abdominal belt for up to four weeks, no extension; flexion, external/internal rotation, and abduction/adduction unrestricted. Knee: flexion restricted due to pain; extension and external/internal rotation unrestricted. After 3 to 4 weeks, we commenced with physiotherapy for the temporomandibular joint, including passive and active exercises for mouth opening, laterotrusion, and mediotrusion. The patients were trained for continuing with the exercises after discharge from hospital.

Postoperative radiographs of the hip and knee were taken one to two weeks after surgery for forensic reasons only. The total hospital stay in our cohort was 15 days for the patient with Goldenhar syndrome, 17 days for the patients with osteomyelitis and adenocarcinoma of the parotid gland, and 21 days for the patient with the odontogenic keratocyst.

## 3. Results

A follow-up from at least 24 months up to 70 months after surgery showed the achievement of a stable occlusion and satisfactory masticatory function with unrestricted mouth opening (interincisal distance of 35–40mm, mean 37.5 mm, Table 1) and preserved or regained lateral and medial excursions in all patients. Fortunately, no flap-related complications, such as necrosis, were observed. Control CT scans showed good bony stabilization and union in all patients. The DCIA allowed for a bony reconstruction anatomically resembling a non-atrophied mandibular body.

Donor-site morbidity was consistently low, assessed using the Lower Extremity Functional Scale (LEFS) [7]. This questionnaire evaluates difficulties in everyday life activities with a numerical score from 0 (maximum disability) to 80 (no limitation). The LEFS score was 80 (100%) in each patient (Table 1).

The patients self-evaluated their facial symmetry as good (as described by Xia et al., Table 1) [8]. All patients were screened using the temporomandibular dysfunction (TMD) pain screener and symptom-related questionnaire [9]. It revealed occasional (once per month) pain in the Goldenhar patient when biting hard food and a joint noise without pain in the adenocarcinoma patient but otherwise no complaints (Table 1).

Facial scars were rated using the Patient and Observer Scar Assessment Scale, which shows consistency and interobserver reliability [10]. The scar is rated from 1 (normal skin) to 10 (worst scar) with respect to vascularity, height/thickness, pliability, surface area, and pigmentation by the clinician and with respect to pain, itching, color, stiffness, thickness, and relief by the patient. We yielded overall good results (Table 1). Due to the skin transfer and therefore lacking comparability, we did not rate the Goldenhar and adenocarcinoma patients. The self-evaluation was not possible for the Goldenhar patient.

## 4. Discussion

The term chimera originates from Greek mythology, referring to a creature with parts from different animals. The definition of chimera flaps in surgery has been controversial since its first introduction in the Annals of Plastic Surgery in 1991 [11]. Following the latest definition given by Halloc, our flap combination is correctly described as a fabricated sequential chimeric flap [12].

The LFC flap was first used in 2015 by Wong et al. for scaphoid necrosis [13]. In 2016, our group performed the first replacements of the mandibular condyle, isolated (see Enzinger et al. 2018) [6] and in combination with the DCIA, which is an established standard flap in microvascular surgery and does not need further description.

In our case series of four patients, the combination of the LFC and DCIA proved to be an appropriate method for reconstructing up to half of the mandible and the condylar head and neck. It is suitable in cases where alloplastic joint replacement cannot be used, such as in large defects, in infected sites (e.g., due to osteomyelitis), in young patients, post-irradiation, and in cases of allergies to the prosthesis materials [2,3,4]. It can also be considered as a second-line therapy when infection, resorption, inadequate growth, or material fatigue have led to the failure of other methods [14,15].

It allows for a precise anatomical reconstruction, replacing bone by vital bone and cartilage by vital cartilage. The LFC provides, unlike other flaps, a cartilaginous joint surface. We observed the possibility of laterotrusive and mediotrusive mandibular movements after attaching the lateral pterygoid muscle to the LFC, allowing for a good functional outcome with close to natural masticatory movements. Compared to the medial femoral condyle flap (MFC), the LFC provides a better curvature radius and a more stable cortical bone and therefore became our method of choice in condylar head replacement [13].

Compared to a fibula or scapula graft, the DCIA flap anatomically best matches a non-atrophied mandibular body and is in our opinion thus the method of choice for replacing parts of the mandibular body.

Mandible and TMJ are restored in one step, immediately stabilizing the occlusion. It is known from trauma surgery that the immediate restoration of the ramus height is a very important factor for a good outcome, preventing occlusal changes and tooth movements and enabling prosthetic rehabilitation later on [6,16]. 

Due to the sequential design, only one supplying artery and vein have to be prepared in the recipient site, and the shortness of the LFC pedicle does not lead to a problem.

If an accessory soft tissue transfer is required, a superior epigastric artery perforator flap, a superior lateral genicular artery perforator flap, or a deep inferior epigastric perforator flap can be harvested and added, making our chimeric flap suitable for more extensive resections, e.g., in tumor patients.

To our knowledge, only four case reports of the LFC flap or adjacent tissue flaps of the superior lateral genicular artery have been published, with no data on donor site morbidities [6,13,17,18]. In our series and other observations at our hospital, donor site morbidity in the LFC harvesting area seems to be low when splitting and not cutting the iliotibial tract and when harvesting from the lateral anterior pole of the knee, which is not load-bearing [6]. 

The necessity of harvesting two flaps leads to an increased operating time and an increased burden of care. Therefore, a careful evaluation of the patient in all biopsychosocial aspects and discussion in the medical team and with the patient is mandatory before going into surgery. The chimeric LFC and DCIA flap method is reserved for maxillofacial surgeons who have already gained significant experience in the field of microsurgery. Further investigations and follow-up studies are required, ensuring the best long-term outcome for our patients and a benefit compared to other reconstructive methods.

## 5. Conclusions

The sequential chimeric LFC and DCIA flap is an appropriate method for reconstructing up to half of the mandible and the condylar head and neck. It is suitable in cases where alloplastic joint replacement cannot be used or where other methods have failed. The LFC provides, unlike other flaps, a cartilaginous joint surface, which allows for both free mouth opening and latero- and mediotrusion. The DCIA flap enables an anatomical reconstruction of the mandibular body. Due to the necessity of harvesting two flaps and the associated increased burden of care, a careful indication is required. Our technique is reserved for maxillofacial surgeons who have already gained significant experience in the field of microsurgery. We hope that it will contribute to an improved care for patients after extended mandibular resections.

## Figures and Tables

**Table 1:** Diagnoses and outcomes in the four patients included in this study.

Patient	Age ^1^	Sex	Primary Diagnosis	LEFS ^2^	TMD Symptoms ^3^	Symmetry Self Evaluation ^4^	Scar Evaluation ^5^	Scar self Evaluation ^5^	MMO Preop. ^6^	MMO Postop. ^6^	Follow Up Time (Month)	Skin Perforator Flap
Patient 1	59	f	Therapy-refractory osteomyelitis	80	No	9	(Hypo)pigmentation 3, relief 4, all others 0	Nearly no complaint	5 mm	40 mm	52	No
Patient 2	63	m	Extended recurrent odontogenic keratozyst	80	No	10	Pliability 1, all others 0	No complaint	40 mm	40 mm	50	No
Patient 3	17	f	Goldenhar syndrome	80	Rarely light pain	Cannot be assessed	Not applicable due to skin transfer	Cannot be assessed	20 mm	35 mm	70	Yes
Patient 4	51	m	Adenocarcinoma of the parotid gland	80	Joint noise, no pain	8	Not applicable due to skin transfer	Nearly no complaint	40 mm	35 mm	24	Yes

^1^ At operation; ^2^ Lower Extremity Functional Scale (LEFS); ^3^ Screened with the temporomandibular dysfunction pain screener and symptom related questionnaire; ^4^ Scale of 1 (least) to 10 (best); ^5^ Patient and Observer Scar Assessment Scale (POSAS). ^6^ Maximal interincisal mouth opening.

## Data Availability

Data are contained within the article.
